# Novel Organic Mineral Complex Prevents High-Fat Diet-Induced Changes in the Gut and Liver of Male Sprague-Dawley Rats

**DOI:** 10.1155/2020/8846401

**Published:** 2020-12-17

**Authors:** M. S. Crawford, A. E. Mohr, K. L. Sweazea

**Affiliations:** ^1^School of Life Sciences, Arizona State University, Tempe, Arizona, USA; ^2^College of Health Solutions, Arizona State University, Phoenix, Arizona, USA

## Abstract

Diet-induced obesity and metabolic syndrome are associated with the onset of gastrointestinal diseases, such as hepatic steatosis and gut inflammation. Prior research shows that a proprietary soil-derived organic mineral complex (OMC) prevents hyperglycemia, endotoxemia, and liver injury in rats fed a high-fat diet (HFD) for 10 weeks. The aim of this study was to further examine the effects of OMC on the liver and gastrointestinal health of these rats. Six-week-old male Sprague-Dawley rats (*n* = 36) were divided into two dietary groups: Chow or HFD fed for 10 weeks. Animals were further divided (*n* = 6/group) and administered 0, 0.6, or 3.0 mg/mL OMC in their drinking water. The 10-week HFD resulted in significant liver fat accumulation. Both OMC doses prevented hepatic increases in the glycation end product N*ε*-(carboxymethyl)lysine (CML) induced by HFD (*p* < 0.05). Low-dose OMC was associated with higher expression of occludin in the small intestine of rats fed either diet (two-way ANOVA, *p* < 0.042). Linear discriminant analysis (LDA) effect size (LEfSe) indicated significant differences in fecal microbial composition of untreated HFD-fed rats in comparison to untreated Chow rats at 10 weeks (LDA score > 2.0 : 18). After 10 weeks, untreated HFD-fed rats were also more abundant in bacteria associated with obesity and metabolic disease in comparison to corresponding week 0 samples (LDA score > 2.0 : 31), 10-week untreated Chow (LDA > 2.0 : 18), or 10-week OMC-treated HFD-fed rats (0.6 mg/mL; LDA > 2.0 : 80, 3.0 mg/mL; LDA > 2.0 : 8). Low-dose OMC prevented the HFD-induced increase in the Firmicutes-to-Bacteroidetes (F/B) ratio (*p* < 0.0416). Study animals treated with OMC exhibited no significant changes in the gut microbiota at week 10, although gut inflammatory biomarkers were not significantly altered by diet or OMC treatment. These results indicate that OMC supplementation ameliorates glycosylation reactions and modifies HFD-induced alterations in the intestinal microbiota.

## 1. Introduction

Metabolic syndrome is characterized by a cluster of pathologies consisting of hypertension, insulin resistance, abdominal obesity, and dyslipidemia [1]. Obesity further contributes to the development of a multitude of hepatic and gastrointestinal (GI) disorders including nonalcoholic fatty liver disease (NAFLD) [2]. Characterized by steatosis (excessive buildup of fat), NAFLD is the most prevalent chronic liver disorder in the world [3], affecting over 65 million Americans with an estimated annual financial burden of $103 billion [4]. Concurrent with the rise of obesity and type 2 diabetes [5], the increase in NAFLD is strongly associated with consumption of a Westernized diet [6]. Hallmarked by high-fat and low-dietary fiber intake, this dietary pattern also modulates the gut microbiome, increasing intestinal dysbiosis, gut wall permeability, and translocation of microbial products (e.g., endotoxins from Gram-negative bacteria) into the circulation [7–10]. These events can ultimately initiate and sustain proinflammatory and oxidative cascades that can lead to liver damage and disease [11, 12]. In turn, liver products (e.g., bile acids) can influence the gut microbiota composition and intestinal barrier integrity [13, 14]. Therefore, the so-called “gut-liver axis” is increasingly recognized as an important factor in the etiology of metabolic syndrome and NAFLD [11, 15]. Due to the complexity of this interaction, many open research questions have emerged, particularly in relation to overall diet and dietary components.

Previously, we reported that short-term consumption of a high-fat diet (HFD; 60% kcal fat) by male Sprague-Dawley rats for 6 weeks promoted simple steatosis through an increase of hepatic lipid accumulation [16]. Furthermore, hepatic injury, lipopolysaccharide (LPS) concentrations, and cecal nuclear factor kappa B (NF-*κ*B) and interleukin-1*β* (IL-1*β*) expression were significantly increased in HFD rats [16]. Additionally, we found that 6-week HFD increased the abundance of Gram-positive bacteria, which has been associated with metabolic disease [16]. A similar study by Jensen et al. indicated that 16 weeks of HFD (60% fat, 20% carbohydrate) consumption resulted in extensive steatosis and inflammation in male Sprague-Dawley rats [5]. Moreover, hepatic dysfunction was confirmed in these HFD-fed rats along with increased levels of liver triacylglycerol and inflammatory markers [5]. Together these findings suggest that an HFD can have profound deleterious effects on liver and GI health.

A first-line treatment recommended for NAFLD and the traditional treatment for obesity and associated comorbidities are dietary intervention [17]. Unfortunately, the success of these interventions is transient, as weight regain and regression to previous health status and poor dietary habits are well-characterized [18]. Therefore, it is important to identify more effective strategies for prevention and treatment of metabolic syndrome and NAFLD. Natural food products and their active components may offer achievable alternatives for the treatment of NAFLD with few adverse effects. The use of natural products and herbal medicines for the management of metabolic diseases continues to rise in popularity in Western societies [19]. Prior research found that a soil-derived mineral extract decreased weight gain, blood glucose, and glycated hemoglobin in a diabetic animal model [20]. Additionally, a recent study from our laboratory demonstrated that an organic mineral complex (OMC) derived from plant and soil fractions prevented symptoms of metabolic syndrome and liver injury (evidenced by lower ALT activity) and mitigated the rise in circulating endotoxin levels induced by consumption of a 10-week HFD in male Sprague-Dawley rats [21].

OMC is a complex of minerals, trace elements, organic acids (particularly fulvic acid), nitrates, and various microbial degradation products from plant and animal origins. Fulvic acid is notable as it has been shown to reduce the release of proinflammatory mediators after exposure to LPS in human monocytes [22] and alter the microbiota of soil ultimately promoting nutrition uptake by plants [22]. However, very little is known about whether these soil-derived products affect the composition and function of the gut microbiota.

Therefore, the objective of the present study was to further investigate the effects of OMC on the gut-liver axis of these animals by determining its effects on HFD-induced pathophysiological changes and gut microbiome composition in six-week-old male Sprague-Dawley rats. We hypothesized that OMC supplementation would mitigate risk factors associated with hepatic and GI inflammation induced by a 10-week HFD.

## 2. Methods and Materials

### 2.1. OMC Isolate

The current study used a proprietary OMC obtained from the study sponsor Isagenix International, LLC (Gilbert, AZ). A compositional analysis of OMC has been described previously [21]. Briefly, OMC is a dietary supplement extracted from several mineral mines in North America and is isolated and manufactured by Mineral Biosciences, LLC (Goodyear, AZ). Similar to the natural substance, shilajit, OMC is composed of over 50 minerals (predominantly calcium, sulfur, potassium, sodium, and magnesium), nitrates, and fulvic acid [21].

### 2.2. Animal Models

This study is a continuation of published work from our laboratory using six-week-old male Sprague-Dawley rats (140–160 g, *n* = 36) randomly divided into two groups that received either standard Chow diet (Teklad Global, 24% protein, 58% carbohydrate, and 18% fat (in % kcal), Indianapolis, IN) or HFD containing 20% protein, 20% carbohydrate, and 60% kcal from fat (Cat. No. D12492, Research Diets Inc., New Brunswick, NJ) for 10 weeks [21]. Rats in each group were administered 0 (control), 0.6, or 3.0 mg/mL OMC in their drinking water throughout the study. Food and OMC-treated water were replaced every 2-3 days to prevent spoiling. Animals were allowed free access to water and food *ad libitum*. At the end of the 10-week study, animals were euthanized (sodium pentobarbital, 200 mg/kg, i.p.) [21]. All procedures were approved by the Arizona State University Institutional Animal Care and Use Committee.

### 2.3. Western Blot Analyses

Proinflammatory cytokines (NF-*κ*B (p65) and IL-1*β*) and the tight junction protein occludin were examined in the cecum and proximal small intestine of all study animals via western blot analyses. Tissue samples were cleaned with ice-cold PBS, pH 7.4 then homogenized in ice-cold Tris-HCl buffer containing 10 mM Tris (pH 7.6), 1 mM EDTA, 1% triton X-100, 0.1% Na-deoxycholate, 0.03% protease phosphatase inhibitor cocktail (Halt Protease and Phosphatase Inhibitor Cocktail, ThermoFisher Scientific, Waltham, MA, USA), and 1 mM phenylmethanesulfonyl fluoride (PMSF) using the BeadBug Microtube Homogenizer for three minutes per the manufacturer's recommendation (3.00 mm zirconium beads, Benchmark Scientific, Edison, NJ, USA). Tissue homogenates were centrifuged at 4000 g for 10 minutes at 4°C to remove insoluble debris. Protein concentrations of the supernatant (soluble proteins) and pellets (membrane-bound proteins) were determined using the Bradford method (Bio-Rad, Hercules, CA). Pellets (occludin) and supernatants (inflammatory cytokines) (50 *μ*g/lane) were resolved by 7.5% Tris-HCl sodium dodecyl sulfate polyacrylamide gel electrophoresis at 200 mV for 35 minutes (SDS-PAGE) (Bio-Rad) and then transferred at 100 mV for 90 min onto polyvinylidene difluoride (PVDF) membranes prewetted with Tris-buffered saline (Bio-Rad, #170-6453) and 0.1% Tween 20 (Bio-Rad, #170-6531), TTBS. Protein molecular weight for phosphorylated NF-*κ*B (p65) was visualized with SeeBlue Plus2 Prestained Standard (ThermoFisher Scientific, Cat. No. LC5925). Protein molecular weight for IL-1*β* and occludin was visualized with ColorBurst Prestained Standard (ThermoFisher Scientific, Cat. No. LC592).

To prevent nonspecific binding of antibodies, the membranes were incubated overnight at 4°C in blocking buffer, TTBS containing 3% bovine serum albumin fraction V (BSA) and 5% nonfat dry milk. The PVDF membranes (Bio-Rad, #160-0174) were then washed with TTBS (3 washes, 5 minutes each) and incubated for 4 hours at room temperature with 1 : 1000 anti-NF-*κ*B p65 rabbit polyclonal antibody (GeneTex, Inc., #GTX11742, Irvine, CA, USA), anti-IL-1*β* mouse monoclonal antibody (Cell Signaling Technologies, #12242, Danvers, MA, USA), or recombinant antioccludin rabbit polyclonal antibody (Abcam, ab167161, Cambridge, MA, USA). Subsequently, membranes were washed in TTBS (5 washes, 5 minutes each) and then incubated with either 1 : 5000 anti-rabbit IgG HRP-linked secondary antibody (NF-*κ*B and occludin) or 1 : 5000 anti-mouse IgG HRP-linked secondary antibody (IL-1*β*), as appropriate, prepared in TTBS for 1 hour at room temperature. Membranes were washed with Tris-buffered saline (TBS, Bio-Rad, 170-6435) after incubation with secondary antibody then exposed to Pierce-enhanced chemiluminescence (ECL) western blotting substrate for one minute (Thermo Scientific, Catalog #32106). The immunoreactive bands were visualized by exposure to X-ray film (Kodak X-OMAT, ThermoFisher Scientific). Total protein concentration was normalized and quantified by staining the PVDF membrane with Coomassie Brilliant Blue R-250 (Bio-Rad, 161-0400) prepared in 50% methanol, 42.5% deionized water, and 7.5% acetic acid. Densitometry was determined using NIH ImageJ software [23].

### 2.4. Oil Red O Staining

Samples of liver tissue were embedded in OCT compound, frozen in isopentane cooled by dry ice, and stored at −80°C until analysis. Lipid droplets and neutral fat were stained using a commercially available Oil Red O staining kit according to the manufacturer's protocol (ScyTek Laboratories, Inc., Logan, UT, USA). Briefly, tissue sections (12 *μ*m) were collected onto color frost microscope slides (VWR, Radnor, PA, USA) using a cryostat (Leica Biosystems CM1950, Buffalo Grove, IL, USA). Sections were incubated with propylene glycol for 5 mins at room temperature followed by heated Oil Red O solution for 10 min. Tissue sections were then treated with 85% propylene glycol for 1 min and rinsed with distilled water. Mayer's hematoxylin (Lillie's modification) was used to counterstain sections, and slides were cover slipped using aqueous mounting medium (Cat No. AML060, ScyTek Laboratories, Inc.), following washes in tap water and distilled water. Images of slides were collected at 40x magnification using an EVOS FL Auto Imaging System from Life Technologies (AMAFD1000, Carlsbad, CA, USA).

### 2.5. Liver Triacylglycerol Assay

Liver triacylglycerol concentrations were measured according to the methods of Jouihan [24]. Briefly, 100–300 mg tissue (*n* = 5 rats per group) was digested in 350 *μ*L ethanolic KOH (1 : 2 ratio of 30% potassium hydroxide and ethanol) overnight at 55°C. The total volume was adjusted to 1000 *μ*L with 50% ethanol, and the mixture was vortexed, then centrifuged at 13,000 rpm for 5 mins. The supernatant was transferred to a clean microcentrifuge tube, and the volume adjusted to 1200 *μ*L with 50% ethanol, then vortexed. A 200 *μ*L aliquot was transferred to a new tube to which 215 *μ*L 1 M magnesium chloride was added, and the mixture was vortexed and then placed on ice for 10 minutes. After centrifuging for 5 minutes at 13,000 rpm, the supernatant was transferred to a new tube to be used for the assay. Liver free glycerol concentrations were determined using a commercially available kit per the manufacturer's protocol (Sigma Aldrich, St. Louis, MO, USA) and triacylglycerol content was determined from the free glycerol concentrations using the following equation: triacylglycerol (mg/g tissue) was calculated as follows: [glycerol] (mg/dl) ∗ (10/30) ∗ (415/200) ∗ 0.012 (dL)/tissue mass (g).

### 2.6. Liver N*ε*-(Carboxymethyl)lysine (CML) Assay

To assess protein glycation, liver CML concentrations (*n* = 6 per treatment group) were measured by homogenizing 100–200 mg tissue in PBS (pH 7.4) (ratio 1 : 9) and 0.03% protease inhibitor cocktail (Sigma Aldrich, P2714). Samples were subjected to three freeze-thaw cycles (freeze overnight at −20°C). The homogenates were then centrifuged for 5 minutes at 5000 × g. Liver CML concentrations were determined using a commercially available ELISA kit per the manufacturer's protocol (Cat No. CY-8066, MBL International, Woburn, MA, USA).

### 2.7. Fecal Microbiome Analyses

#### 2.7.1. Fecal Microbial DNA Preparation

Microbial genomic DNA isolation was performed using the PowerSoil® DNA Isolation Kit (MoBio Laboratories, Inc., Carlsbad, CA, USA) per the manufacturer's protocol. DNA concentration was quantified using a *μ*Drop™ plate adaptor (Catalog #N12391, ThermoFisher Scientific, Waltham, MA, USA) and Multiskan™ GO microplate spectrophotometer (Catalog #5119300, ThermoFisher Scientific Waltham, MA, USA). Samples used for analysis were required to have a purity of OD_260_/OD_280_ ratio of 1.8.

#### 2.7.2. Illumina Sequencing and Microbial Sequence Analyses

The Microbiome Analysis Laboratory at Arizona State University implemented QIIME analyses and Illumina sequencing to analyze microbial taxonomy from week 0 and week 10 fecal samples as previously described [21, 25]. Subsequently, microbial differences were examined using linear discriminant analysis of effect size (LEfSe) analyses implemented within the online Galaxy module (http://huttenhower.sph.harvard.edu/galaxy/) explained by Segata et al. [25]. Relative abundance percentages were then calculated from taxonomic data generated from QIIME.

## 3. Statistical Analyses

Data are expressed as means ± SEM. Data were analyzed using SigmaPlot (Systat Software Version 10.0, San Jose, CA, USA). Western blot, liver triacylglycerol/glycerol, and CML data were analyzed by two-way ANOVA with diet (Chow and HFD) and treatment (dose of OMC) as factors. Average relative abundance of phyla was calculated by taking the individual phylum absolute abundance per sample, dividing by the sample sum of absolute abundances, then transformed to a percentage. Relative abundance data were analyzed by one-way ANOVA followed by Bonferroni *post hoc* tests. Week 0 and Week 10 Firmicutes-to-Bacteroidetes ratios were calculated by dividing the relative abundance of Firmicutes by that of Bacteroidetes for all groups and analyzed by two-way ANOVA followed by Tukey *post hoc* tests. Statistical significance was considered for *p* < 0.05 for all analyses. LEfSe analysis was conducted using Kruskal–Wallis sum-rank tests, Wilcoxon rank-sum tests, and linear discriminant analysis as previously described [25].

## 4. Results

### 4.1. Western Blot Analyses

#### 4.1.1. Proinflammatory Protein Expression in the Cecum and Small Intestine

**Figure 1:**
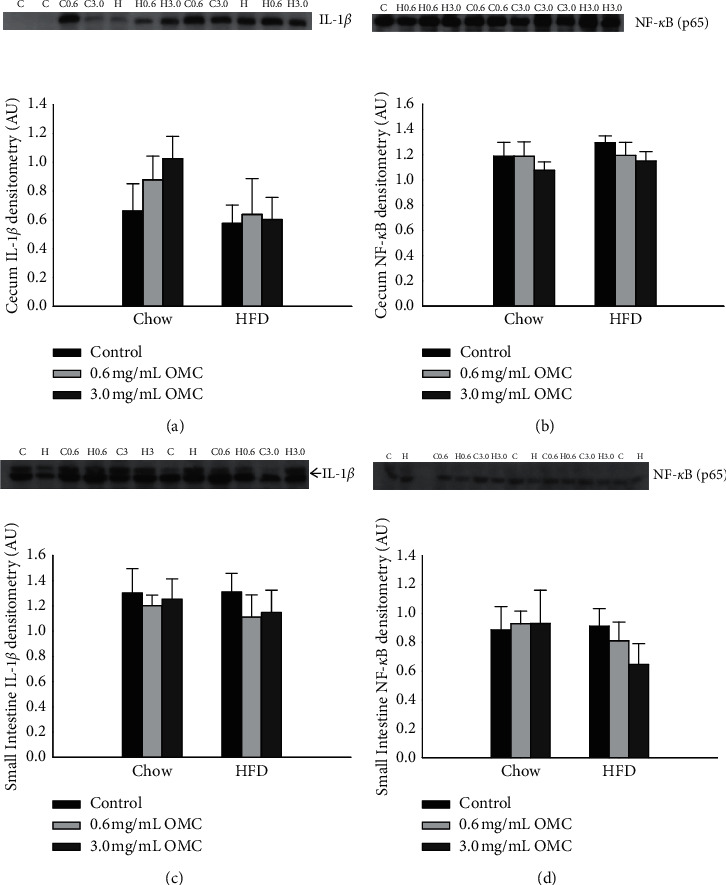
Protein expression of inflammatory cytokines in the cecum and small intestine. (a) IL-1*β* protein expression in cecum homogenates from rats in all treatment groups (*n* = 6/group), *p*=0.702. Effects of OMC supplementation on IL-1*β* expression, *p*=0.602. HFD vs OMC interaction, *p*=0.783. (b) NF-*κ*B (p65) protein expression in cecum homogenates from rats in all treatment groups (*n* = 6/group), *p*=0.362. Effects of OMC supplementation on NF-*κ*B expression, *p*=0.803. HFD vs OMC interaction, *p*=0.855. (c) IL-1*β* protein expression in small intestine homogenates from rats in all treatment groups (*n* = 6/group), *p*=0.603. Effects of OMC supplementation on IL-1*β* expression, *p*=0.574. HFD vs OMC interaction, *p*=0.931. (d) NF-*κ*B (p65) protein expression in small intestine homogenates from rats in all treatment groups (*n* = 6/group), *p*=0.746. Effects of OMC supplementation on NF-*κ*B expression, *p*=0.304. HFD vs OMC interaction, *p*=0.586. Densitometry values were normalized to Coomassie for all samples, and data are expressed as mean ± SEM and analyzed by two-way ANOVA.

10-week HFD consumption did not affect the expression of the proinflammatory cytokine IL-1*β* (the cecum: Figure 1(a); *df* = 1, *F* = 3.744, and *p*=0.062; the small intestine: Figure 1(c); *df* = 1, *F* = 0.277, and *p*=0.603) or transcription factor NF-*κ*B (the cecum: Figure 1(b); *df* = 1, *F* = 0.723, and *p*=0.402; the small intestine: Figure 1(d); *df* = 2, *F* = 0.296, *p*=0.746). Similarly, OMC supplementation had no effect on expression of NF-*κ*B (the cecum: Figure 1(b); *df* = 2, *F* = 1.050, and *p*=0.362; the small intestine: Figure 1(c); *df* = 1, *F* = 1.096, and *p*=0.304) or IL-1*β* (the cecum: Figure 1(a); *df* = 2, *F* = 0.358, and *p*=0.702; the small intestine: Figure 1(c)*df* = 2, *F* = 565, and *p*=0.574). Moreover, there were no significant interactions between HFD and OMC supplementation (the cecum: NF-*κ*B, *df* = 2, *F* = 0.246, and *p*=0.855; IL-1*β*, *df* = 2, *F* = 0.246, and *p*=0.783; the small intestine: NF-*κ*B, *df* = 2, *F* = 0.545, and *p*=0.586; IL-1*β*, *p*=0.931).

#### 4.1.2. Tight Junction Protein Expression in the Cecum

**Figure 2:**
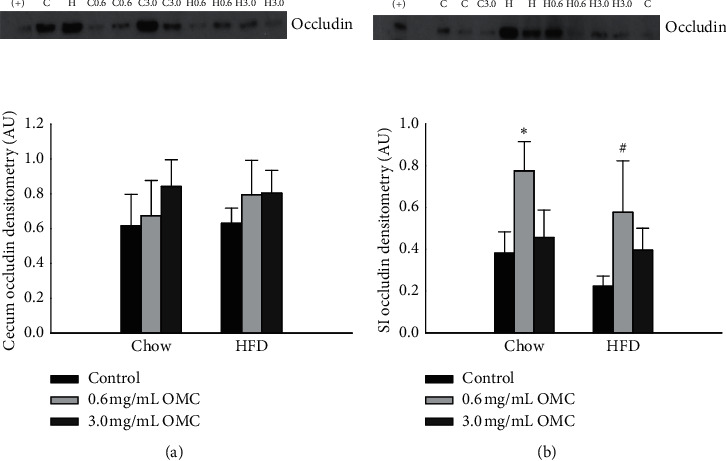
Occludin protein expression in the cecum and small intestine (SI). (a) Occludin protein expression in cecum homogenates from rats in all treatment groups (*n* = 5-6/group), *p*=0.686. Effects of OMC supplementation on occludin expression, *p*=0.605. Diet vs dose interaction, *p*=0.943. (b) Occludin protein expression in small intestine homogenates from rats in all treatment groups (*n* = 5-6/group), *p*=0.259. Effects of OMC supplementation on occludin expression, ^*∗*^*p* < 0.05 Chow 0.6 mg/mL OMC vs Chow, and ^#^*p* < 0.05 HFD 0.6 mg/mL OMC vs HFD. Diet vs dose interaction, *p*=0.891. Densitometry values were normalized to Coomassie for all samples. Data are expressed as mean ± SEM and analyzed by two-way ANOVA.

10-week HFD consumption did not affect the expression of the tight junction protein occludin in the cecum (the cecum: Figure 2(a); *df* = 1, *F* = 0.166, and *p*=0.686; the small intestine: Figure 2(b)*df* = 1, *F* = 1.330, and *p*=0.259). Additionally, OMC supplementation did not influence the expression of occludin in the cecum (*df* = 2, *F* = 0.512, and *p*=0.605). In contrast, low-dose OMC supplementation increased protein expression of occludin in the small intestine of both Chow- and HFD-fed animals (*df* = 2, *F* = 3.421, and *p*=0.047).

### 4.2. Liver CML

**Figure 3:**
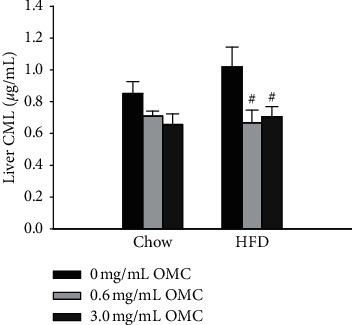
Liver N*ε*-(carboxymethyl)lysine concentrations. Data are expressed as mean ± SEM and analyzed by two-way ANOVA, *n* = 6/treatment group, ^#^*p* < 0.05 vs. HFD untreated.

There was no significant difference between 10-week HFD and Chow groups in liver CML concentrations (Figure 3; *df* = 1, *F* = 0.822, and *p*=0.372). However, administration of either dose of OMC (0.6 mg/mL and 3.0 mg/mL) resulted in lower liver CML compared to HFD animals not treated with OMC (Figure 3; 0.00 vs 0.6 mg/mL, *p*=0.01; 0.00 vs 3.0 mg/mL, *p*=0.008).

### 4.3. Liver Free Glycerol and Triacylglycerol

**Figure 4:**
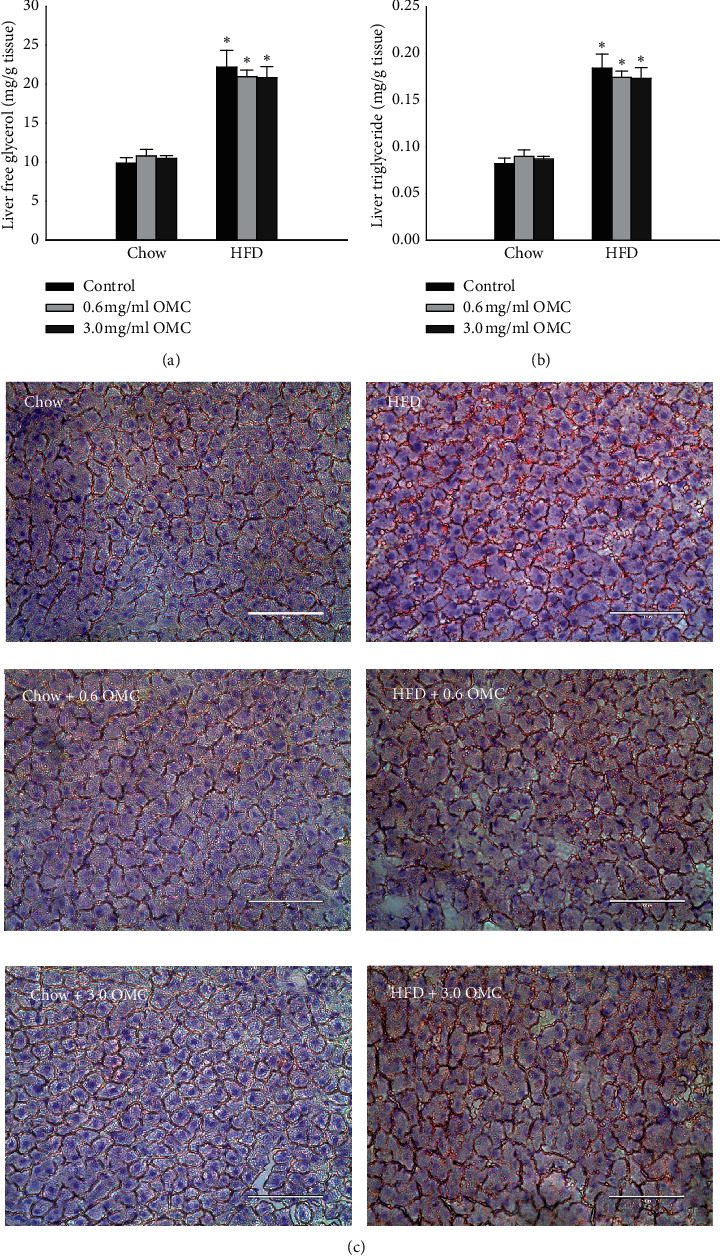
Hepatic lipid accumulation. (a) Free glycerol and (b) triacylglycerol concentrations in liver samples isolated from *n* = 5 animals in each group. Data are expressed as mean ± SEM and analyzed by two-way ANOVA, ^*∗*^*p* < 0.05 vs respective Chow control. (c) Representative images of Oil Red O stained hepatic tissues collected from male Sprague-Dawley rats in each treatment group. Frozen tissue sections were stained with Oil Red O to show neutral triglycerides and lipid content and counterstained with hematoxylin to show nuclei (blue). Livers from rats fed HFD for 10 weeks showed evidence of simple steatosis.

Liver free glycerol and triacylglycerol concentrations increased significantly following the consumption of HFD compared to Chow (Figures 4(a) and 4(b); *df* = 1, *F* = 134.7, and *p* < 0.001). OMC supplementation did not have an effect. Additionally, Oil Red O staining of liver sections showed 10-week HFD increased hepatic neutral triglycerides and lipids indicative of simple steatosis (Figure 4(c)). Neither dose of OMC (0.6 mg/mL and 3.0 mg/mL) prevented HFD-induced steatosis (Figure 4).

### 4.4. Fecal Microbiome Analyses

#### 4.4.1. Linear Discriminant Analysis Effect Size (LEfSe)

**Figure 5:**
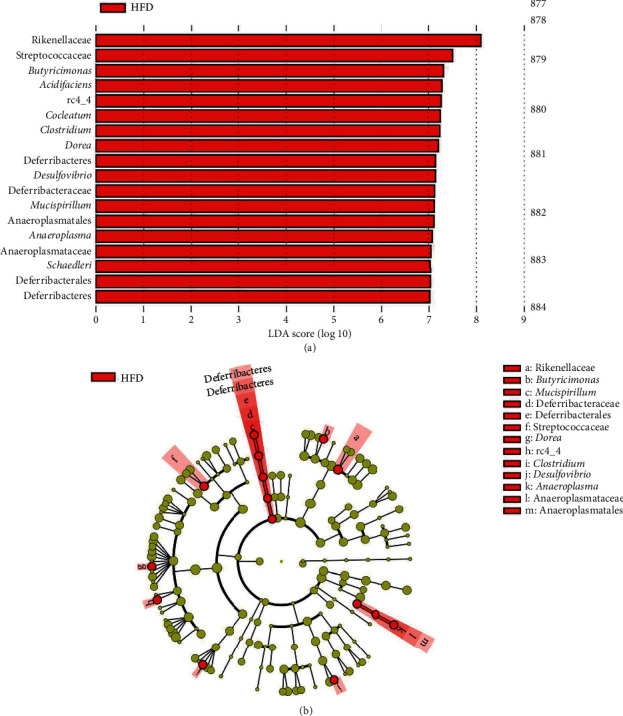
Differently abundant taxa after 10 weeks of either Chow or HFD. (a) Linear discriminate analysis of effect size (LEfSe) results depicting different microbial taxonomic signatures in fecal samples collected from male Sprague-Dawley rats fed an HFD (*n* = 5-6/group) at week 10 in comparison to Chow-fed samples at week 10. Fecal samples collected from HFD-fed rats were more abundant in the following phyla: Bacteroidetes, Firmicutes, *Clostridium*, Deferribacteres, Proteobacteria, and Tenericutes (LDA > 2.0 : 18). No other differences were detected. (b) Cladogram depicting differentially abundant microbial taxa in stool samples collected from male Sprague-Dawley rats fed a high-fat diet (*n* = 5-6) for 10 weeks. Microbial taxa enriched in HFD rats are shown in red.

**Figure 6:**
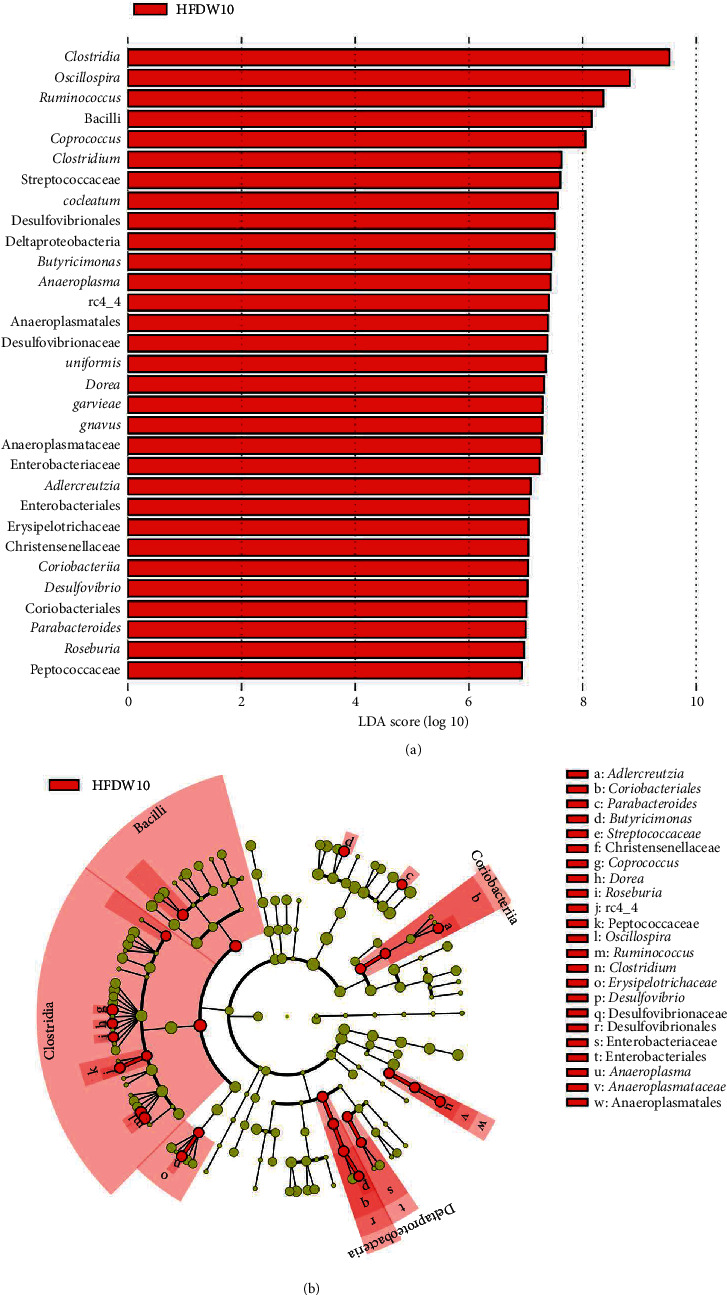
Change in differently abundant taxa between fecal samples of HFD rats collected at week 0 and week 10. (a) Linear discriminate analysis of effect size (LEfSe) results depicting different microbial taxonomic signatures in fecal samples collected from male Sprague-Dawley rats fed HFD (*n* = 5-6/group) at week 10 in comparison to HFD week 0 samples. Fecal samples collected from HFD-fed rats at week 10 were more abundant in bacteria in the following phyla: Firmicutes, Proteobacteria, Bacteroidetes, Tenericutes, and Actinobacteria. No other differences were detected. (b) Cladogram depicting differentially abundant microbial taxa in stool samples collected from male Sprague-Dawley rats fed HFD (*n* = 5-6/group) for 10 weeks. Microbial taxa enriched in HFD rats are shown in red.

**Figure 7:**
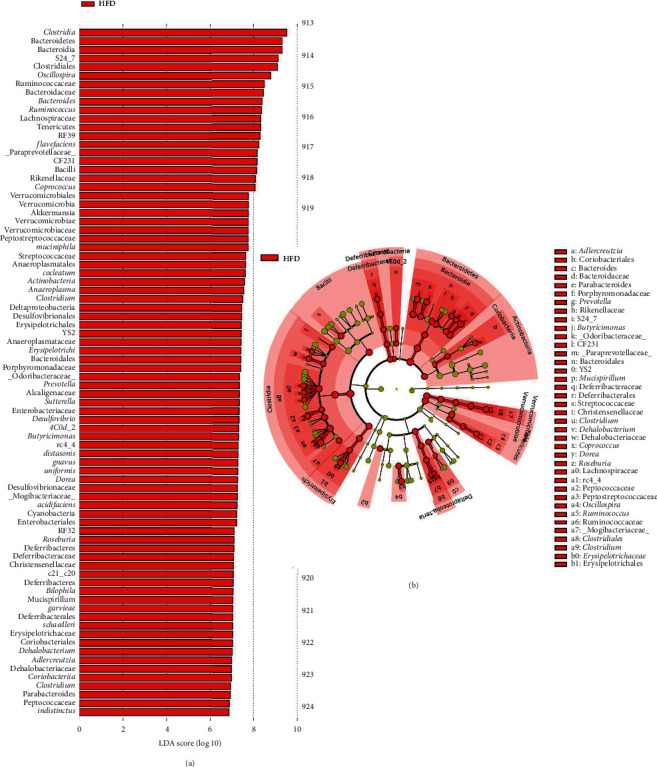
Effects of low-dose OMC on taxa abundance in rats fed HFD for 10 weeks. (a) Linear discriminate analysis of effect size (LEfSe) results depicting different microbial taxonomic signatures in fecal samples collected from male Sprague-Dawley rats fed a 10-week HFD (*n* = 6/group) in comparison to HFD-fed rats treated with 0.6 mg/mL OMC for 10 weeks. No other differences were detected. (b) Cladogram depicting differentially abundant microbial taxa in stool samples collected from male Sprague-Dawley rats fed HFD (*n* = 5-6/group) for 10 weeks. Microbial taxa enriched in HFD rats are shown in red.

**Figure 8:**
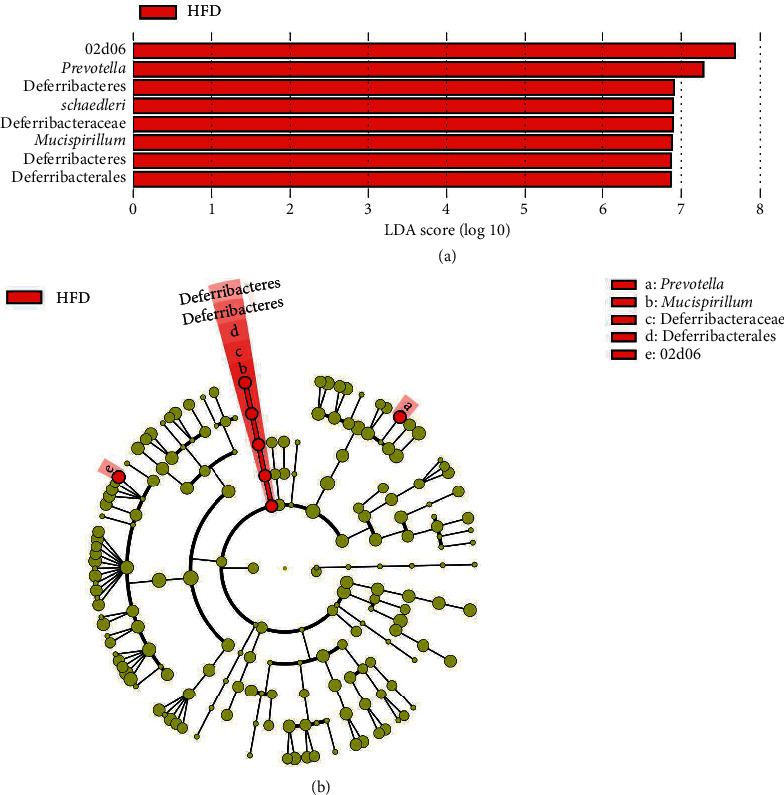
Effects of high-dose OMC on taxa abundance in rats fed HFD for 10 weeks. (a) Linear discriminate analysis of effect size (LEfSe) results depicting different microbial taxonomic signatures in fecal samples collected from male Sprague-Dawley rats fed a 10-week HFD (*n* = 5-6/group) in comparison to HFD-fed rats treated with 3.0 mg/mL OMC for 10 weeks. No other differences were detected. (b) Cladogram depicting differentially abundant microbial taxa in stool samples collected from male Sprague-Dawley rats fed an HFD (*n* = 5-6/group) for 10 weeks. Microbial taxa enriched in HFD rats are shown in red.

LEfSe analyses indicated significant differences in fecal microbial taxonomy in animals fed HFD in comparison to the Chow control (Figure 5; LDA > 2.0 : 18 clades). Fecal samples from HFD rats at 10 weeks were more abundant in the following phyla: Bacteroidetes, Firmicutes, *Clostridium*, Deferribacteres, Proteobacteria, and Tenericutes. When compared to the animal's week 0 samples, a 10-week HFD increased the abundance of Firmicutes, Proteobacteria, Bacteroidetes, Tenericutes, and Actinobacteria (Figure 6; LDA score > 2.0 : 31 clades). Additionally, at 10 weeks, the microbial composition of fecal samples from untreated HFD-fed rats was significantly different from that of HFD-fed study animals treated with 0.6 mg/mL (Figure 7; LDA > 2.0 : 80 clades) and 3.0 mg/mL OMC (Figure 8; LDA > 2.0 : 8 clades).

#### 4.4.2. Average Percent Relative Abundance

**Figure 9:**
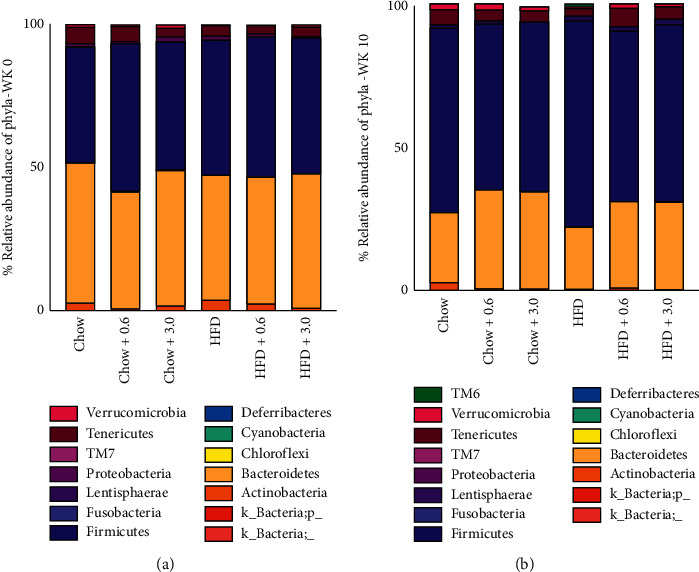
Average percent relative abundance of phyla. (a) Relative abundance of microflora at phylum level in fecal samples of each group at week 0. Data were analyzed by one-way ANOVA followed by Bonferroni *post hoc* test found no significant differences between groups (*n* = 5-6/group). 14 phyla were identified. (b) Relative abundance of microflora at phylum level in fecal samples of each group at week 10. Data were analyzed by one-way ANOVA followed by Bonferroni *post hoc* test found no significant differences between groups (*n* = 5-6/group). 15 phyla were identified.

Analysis of the average percent relative abundance of week 0 and week 10 fecal samples found no significant differences between groups (Figure 9, Supplemental Tables 1 and 2).

#### 4.4.3. Firmicutes-to-Bacteroidetes Ratio

**Figure 10:**
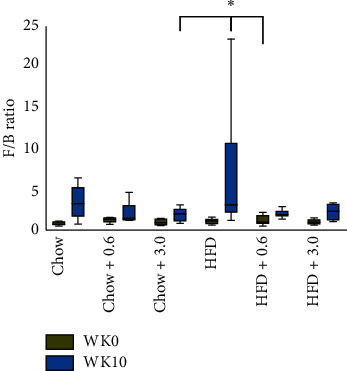
Firmicutes-to-Bacteroidetes ratio of all groups at week 0 and week 10. Data were analyzed by two-way ANOVA RM followed by Tukey *post hoc* test, ^∗^denotes *p* < 0.0355 and *p*=0.0416 compared to Chow + 3.0 mg/mL and HFD + 0.6 mg/mL F/B ratio at week 10, respectively (*n* = 5-6/group).

The Firmicutes : Bacteroidetes ratio was calculated as a marker of gut dysbiosis. The F/B ratio was lower in fecal samples from HFD-fed rats treated with 0.6 mg/mL OMC as compared to untreated HFD rats (Figure 10; two-way ANOVA, Tukey *post hoc* test, *p*=0.0416). Additionally, “time” was a significant source of variation (two-way ANOVA, *df* = 1, *F* = 9.932, and *p*=0.0038).

## 5. Discussion

With the increasing incidence of gastrointestinal and metabolic complications in Westernized countries, natural health products are increasingly being considered as alternative treatments for these diseases. Indeed, gut microbiota-targeted therapies have been suggested for NAFLD, including the use of probiotics, prebiotics, antibiotics, and some active components found in herbal medicines [26]. A prior study from our laboratory demonstrated that OMC prevents HFD-induced increases in circulating LPS, a marker of gut integrity [21]. Based on this observation, the goal of the present study was to examine the effects of OMC on HFD-induced gastrointestinal and liver pathophysiological outcomes in these rats. A potential limitation of the present study is the relatively small sample size, which reflects the research design of the original work [21] and is consistent with sample sizes included in a similar study examining the effects of a 6-week HFD on the gut microbiome [16].

The results from the current study show that (1) a 10-week HFD induced gut dysbiosis, as well as hepatic steatosis and accumulation of advanced glycation end products (CML) in young male Sprague-Dawley rats; (2) although OMC did not prevent hepatic steatosis, it was effective at preventing HFD-induced changes to the gut microbiome and hepatic CML levels; and (3) OMC may help prevent gut leakiness as it promoted the expression of occludin in the small intestine. Together these findings are suggestive of a potential pathological link between the gut microbiota and liver induced by HFD, which may be prevented by supplementation with OMC.

The gut-liver axis is increasingly recognized as an important factor in the development of metabolic syndrome and NAFLD. Key features of this connection include gut wall permeability, translocation of microbial products [7–10], and propagation of proinflammatory and oxidative cascades that can lead to liver damage and disease [11, 12]. Additionally, products from the gut microbiota can impact the liver and, in turn, liver products can influence the gut microbiota composition and gut barrier integrity [13, 14]. The major drivers of increased gut permeability include inflammation and dysbiosis [27, 28], which have been linked to consumption of a high-fat Western diet [9, 29, 30]. Prior studies indicate that high-fat feeding can reduce the expression of tight junction proteins occludin and zona-occludens (ZO)-1 and also increase plasma endotoxemia in C57bl6/J mice [31], whereas protein expression of occludin was not significantly different between groups and low-dose OMC supplementation was associated with higher expression of occludin in the proximal small intestine of both the Chow and HFD-fed rats. Although not analyzed in the current study, this effect may be due to the presence of nitrate in OMC. Nitrate is reduced to nitrite in the oral cavity then nitric oxide (NO) in the stomach [32]. Previous studies have shown that NO may act as a nutrient for the lower microbiome [33] and prevent intestinal barrier dysfunction by protecting tight junction proteins from reactive oxygen species through redox reactions [32, 34]. However, an increase in intestinal permeability facilitates passive translocation of luminal LPS or LPS-containing bacteria into the lamina propria, promoting greater translocation of LPS into blood (termed endotoxemia) [35]. For this reason, circulating LPS concentration is a surrogate marker for assessing *in vivo* intestinal permeability [36]. As our prior study showed OMC prevented HFD-induced increases in circulating LPS [21], it is possible that OMC may similarly strengthen the intestinal barrier through increased expression of occludin.

Prior studies have shown that high-fat feeding is linked to increased expression of proinflammatory cytokines in the gastrointestinal tract [16, 37, 38]. However, in the current study, consumption of an HFD for 10 weeks did not increase the expression of inflammatory markers (NF-*κ*B (p65) and IL-1*β*) in the cecum or proximal small intestine. In studies of C57BL/6 mice, consumption of an HFD for 2–16 weeks only increased TNF-*α* in the ileum [39]. Tumor necrosis factor-*α* is an important inducer of NF-*κ*B (p65) [40], and an earlier study indicated that LPS can activate intestinal NF-*κ*B with the support of neutrophils and endogenous TNF-*α* [41]. However, in the current study, TNF-*α* was undetectable in plasma samples. Thus, it is possible that HFD intake for 10 weeks likewise did not increase intestinal TNF-*α* or activation of NF-*κ*B, although it is possible that HFD-intake altered the expression of other proinflammatory cytokines in the gut or elsewhere in the body. In fact, others have shown that diet-induced obesity and metabolic syndrome are marked by an increase in proinflammatory markers secreted from adipose tissue [42]. In addition, studies have shown that HFD consumption can increase LPS which is translocated from the gut and subsequently participates in the inflammatory responses in adipose tissue [43]. Our prior study likewise showed increased plasma LPS following 10-week HFD in these same study animals [21]. Therefore, it is possible that inflammatory cytokines were altered in adipose tissue, although that was not examined in the present study.

Elevated circulating LPS can accelerate lipid accumulation [44] and promote liver injury [45]. Moreover, hepatic lipid accumulation leads to increased endogenous formation of CML, likely due to increased lipid peroxidation [46] and inflammation [47]. CML levels are reportedly correlated with the severity of liver disease [48], and advanced glycation end products have been suggested to play a critical role in the pathogenesis of NAFLD [49]. One mechanism through which CML affects cellular function is binding to the receptor for AGEs and activating intracellular signaling pathways such as NF-*κ*B [50, 51], which could contribute to the pathogenesis of NAFLD. Findings from the present study showed lower CML levels in HFD rats receiving OMC supplementation without significantly affecting lipid accumulation in the liver. These results are corroborated with our previous findings showing decreased ALT, a marker for liver injury [21]. As the HFD rats displayed increased lipid accumulation in the liver, OMC supplementation may have a protective benefit against steatosis and NAFLD.

The gut microbiome is implicated in the onset of many metabolic and inflammatory disorders. Consistent with prior studies of rodents [21, 52], chronic high-fat feeding significantly increased liver triacylglycerol and altered the gut microbial communities in male Sprague-Dawley rats. HFD-induced pathogenic state results in an imbalance (dysbiosis) of Firmicutes and Bacteroidetes. Interestingly, the F/B ratio of untreated HFD-fed rats at 10 weeks was significantly higher than HFD-fed rats treated with the low dose of OMC. Therefore, it is possible that fulvic acid, the main active component in OMC treatment [21], may prevent an increase in Firmicutes in the study animals. While very little information is known about the effects of fulvic acid, a 2017 study by Gao et al. found that 60-day fulvic acid feeding decreased the abundance of Firmicutes in the intestine of *Paramisgurnus dabryanus* [53]. Additionally, Gao et al. showed that fulvic acid can increase lysozyme levels in the intestine [53], which has been shown to significantly decrease Firmicutes levels in pregnant sows [54].

Results from LEfSe analyses showed differences in taxonomic structure between rats fed a Chow or HFD after 10 weeks. In comparison to Chow-fed rats, fecal samples from untreated HFD-fed rats were more enriched in Firmicutes (rc4_4, *Dorea*, *Clostridium cocleatum*, and *schaedleri*), Deferribacteres (*Desulfovibrio*, Deferribacteraceae, Deferribacterales, Deferribacteres, and *Mucispirillum*), Bacteroidetes (Rikenellaceae, Streptococcaceae, and *acidifaciens*), Proteobacteria (*Desulfovibrio*), and Tenericutes (Anaeroplasmatales, *Anaeroplasma*, and Anaeroplasmataceae) families and species at 10 weeks; all of which are associated with the development of inflammation, obesity, and type 2 diabetes [55–60]. Moreover, fecal samples from these HFD-fed rats were more abundant in bacteria associated with high-fat diet intake from the phyla Bacteroidetes (*Parabacteroides*, a commensal microbe) [61], Firmicutes (*Oscillospira*, *Ruminococcus*, *garvieae*, *gnvaus*, *Roseburia*, and Peptococcaceae), Actinobacteria (Christensenellaceae, Coriobacteriia, and Coriobacteriales), and Proteobacteria (Enterobacteriales) in comparison to fecal samples collected at week 0. LEfSe analysis also revealed 10-week OMC supplementation (0.6 mg/mL and 3.0 mg/mL) prevented alterations in microbial composition induced by HFD-intake. These findings were likely influenced by consumption of energy dense diets, including the ingestion of high amounts of saturated fat [62]. In addition, these data are consistent with our prior work, which revealed that 6 weeks of HFD feeding increased the abundance of Firmicutes, Proteobacteria, Clostridiales, and Streptococcaceae [16].

Several taxa noted in other studies assessing the gut microbiome of humans with NAFLD or cirrhosis had increased abundance, including Streptococcaceae [63], *Dorea* [64, 65], and *Clostridium* [66]. These are notable findings as Streptococcaceae is known to induce persistent inflammation in the intestinal mucosa [67], and increased abundance of Streptococcaceae has been noted in other, similar research studies. For example, in a study that fed germ-free mice an HFD and inoculated them with feces from patients with NASH, the mice had markedly elevated serum levels of ALT, AST, endotoxin, IL-6, monocyte chemotactic protein 1, and hepatic triglycerides [68]. Similarly, increased abundance of *Dorea* has been reported in the gut microbiome of HFD-fed C57BL/6 male mice [68]. In a study assessing the gut microbial changes during liver disease progression using a streptozotocin-HFD induced NASH-HCC C57BL/6J mouse model that is highly relevant to human liver disease, *Clostridium* was found to be positively associated with increased LPS levels [69]. We also noted an increased abundance of the genus *Ruminococcus* induced by 10 weeks of high-fat intake when compared to baseline for these animals (i.e., week 0). In this genus, there are species that produce alcohol [70]. Endogenous ethyl alcohol from the gut can reach the liver by the portal vein, which could contribute to liver damage and aggravate the pathology of NAFLD [71]. Together, the increased abundance of these taxa in the gut microbiome of the HFD rats examined in the present study is suggestive of a state of gut dysbiosis as well as hepatic steatosis and glycation.

Consistent with prior research demonstrating that OMC supplementation may play a vital role in preventing several symptoms of metabolic syndrome [21], OMC was shown to prevent alterations in liver CML and the fecal microbial composition of rats fed HFD for 10 weeks. Due to the paucity of research on the effects of fulvic acid and other components of OMC on the gut microbiota, these results are difficult to explain mechanistically. OMC may be acting directly within the GI tract, as well as exerting indirect effects, such as through the liver. In the liver, fulvic acid can act as an antioxidant by uncoupling electron transport in liver mitochondria leading to a decrease in reactive oxygen species (ROS) production [72], which may explain the previous observation that OMC prevents HFD-induced increases in ALT activity [21].

## 6. Conclusion

In summary, the current study demonstrated that a 10-week HFD alters gut microbial composition and promotes hepatic steatosis; however, it did not elevate markers of gut inflammation. In addition, OMC supplementation prevented changes in CML levels and the gut microbiota, while increasing expression of occludin, a fundamental tight junction protein. This study provides support for further investigation into therapeutic and preventive properties of this natural health product on the alleviation of metabolic disease risk factors in the gastrointestinal and hepatic systems.

## Data Availability

The data used to support the findings of this study are available upon request.

## Supplementary Materials

Supplementary Materials Supplemental Table 1: statistical analyses of week 0 average percent relative abundance of microbiota in fecal samples collected from animals in each group. Supplemental Table 2: statistical analyses of week 10 average percent relative abundance of microbiota in fecal samples collected from animals in each group.
