# Supplementary material for: Novel Organic Mineral Complex Prevents High-Fat Diet-Induced Changes in the Gut and Liver of Male Sprague-Dawley Rats
Source: J Nutr Metab. 2020 Dec 17;2020:8846401. doi: 10.1155/2020/8846401 (PMC7768589; doi:10.1155/2020/8846401)
Supplement: Supplementary Materials — Supplemental Table 1: statistical analyses of week 0 average percent relative abundance of microbiota in fecal samples collected from animals in each group. Supplemental Table 2: statistical analyses of week 10 average percent relative abundance of microbiota in fecal samples collected from animals in each group. [file 8846401.f1.zip › 8846401.f1/Crawford et al., Supplemental Table 1.docx]

**Supplemental Table 1:** *Statistical Analyses of Week 0 Average Percent Relative Abundance of Microbiota in Fecal Samples Collected from Animals in Each Group.*

| **Phyla** |  | **SS** | **DF** | **MS** | **F** | ***p*** |
| --- | --- | --- | --- | --- | --- | --- |
| **k_Bacteria;__** | Treatment | 0.0005694 | 5 | 0.001139 | 0.6867 | 0.6375 |
|  | Residual | 0.04643 | 28 | 0.001658 |  |  |
|  | Total | 0.05213 | 33 |  |  |  |
| **k__Bacteria;p__** | Treatment | 0.0005756 | 5 | 0.0001151 | 0.5609 | 0.7290 |
|  | Residual | 0.005747 | 28 | 0.0002053 |  |  |
|  | Total | 0.006323 | 33 |  |  |  |
| **Actinobacteria** | Treatment | 36.28 | 5 | 7.257 | 0.849 | 0.5267 |
|  | Residual | 239.2 | 28 | 8.543 |  |  |
|  | Total | 275.5 | 33 |  |  |  |
| **Bacteroidetes** | Treatment | 230.2 | 5 | 46.03 | 0.5593 | 0.7301 |
|  | Residual | 2305 | 28 | 82.31 |  |  |
|  | Total | 2535 | 33 |  |  |  |
| **Chloroflexi** | Treatment | 8.590e-006 | 5 | 1.718e-006 | 1.194 | 0.3374 |
|  | Residual | 4.029e-005 | 28 | 1.439e-006 |  |  |
|  | Total | 4.883_005 | 33 |  |  |  |
| **Cyanobacteria** | Treatment | 1.297 | 5 | 0.2593 | 1.198 | 0.3355 |
|  | Residual | 6.060 | 28 | 0.2164 |  |  |
|  | Total | 7.356 | 33 |  |  |  |
| **Deferribacteres** | Treatment | 1.228 | 5 | 0.2456 | 0.779 | 0.5730 |
|  | Residual | 8.826 | 28 | 0.3152 |  |  |
|  | Total | 10.05 | 33 |  |  |  |
| **Firmicutes** | Treatment | 365.5 | 5 | 73.11 | 1.002 | 0.4348 |
|  | Residual | 2043 | 28 | 72.96 |  |  |
|  | Total | 2409 | 33 |  |  |  |
| **Fusobacteria** | Treatment | 4.126e-006 | 5 | 8.252e-007 | 0.9224 | 0.4812 |
|  | Residual | 2.505e-005 | 28 | 8.946e-007 |  |  |
|  | Total | 2.918e-005 | 33 |  |  |  |
| **Lentisphaerae** | Treatment | 8.232e-005 | 4 | 2.058e-005 | 0.9036 | 0.4782 |
|  | Residual | 0.0005238 | 23 | 2.278e-005 |  |  |
|  | Total | 0.0006062 | 27 |  |  |  |
| **Proteobacteria** | Treatment | 5.436 | 5 | 1.087 | 1.648 | 0.1802 |
|  | Residual | 18.48 | 28 | 0.6599 |  |  |
|  | Total | 23.91 | 33 |  |  |  |
| **TM7** | Treatment | 4.358e-005 | 5 | 8.716e-006 | 0.4037 | 0.8420 |
|  | Residual | 0.0006044 | 28 | 2.159e-005 |  |  |
|  | Total | 0.0006480 | 33 |  |  |  |
| **Tenericutes** | Treatment | 34.29 | 5 | 6.857 | 1.208 | 0.3312 |
|  | Residual | 159.0 | 28 | 5.677 |  |  |
|  | Total | 193.3 | 33 |  |  |  |
| **Verrucomicrobia** | Treatment | 4.662 | 5 | 93.25 | 0.3279 | 0.8919 |
|  | Residual | 79.64 | 28 | 2.844 |  |  |
|  | Total | 84.30 | 33 |  |  |  |

One-way ANOVA was used to examine week 0 differences between the means of all groups for each specified phylum including sum of squares (SS), degrees of freedom (DF), mean square (MS), “F” values and significant “*p*” values.
