# Supplementary material for: Novel Organic Mineral Complex Prevents High-Fat Diet-Induced Changes in the Gut and Liver of Male Sprague-Dawley Rats
Source: J Nutr Metab. 2020 Dec 17;2020:8846401. doi: 10.1155/2020/8846401 (PMC7768589; doi:10.1155/2020/8846401)
Supplement: Supplementary Materials — Supplemental Table 1: statistical analyses of week 0 average percent relative abundance of microbiota in fecal samples collected from animals in each group. Supplemental Table 2: statistical analyses of week 10 average percent relative abundance of microbiota in fecal samples collected from animals in each group. [file 8846401.f1.zip › 8846401.f1/Crawford et al., Supplemental Table 2.docx]

**Supplemental Table 2:** *Statistical Analyses of Week 10 Average Percent Relative Abundance of Microbiota in Fecal Samples Collected from Animals in Each Group.*

| **Phyla** |  | **SS** | **DF** | **MS** | **F** | ***p*** |
| --- | --- | --- | --- | --- | --- | --- |
| **k_Bacteria;__** | Treatment | 0.007670 | 5 | 0.001534 | 0.4496 | 0.8099 |
|  | Residual | 0.09553 | 28 | 0.003412 |  |  |
|  | Total | 0.1032 | 33 |  |  |  |
| **k__Bacteria;p__** | Treatment | 0.0003799 | 5 | 7.598e-005 | 0.6872 | 0.6371 |
|  | Residual | 0.003096 | 28 | 0.0001106 |  |  |
|  | Total | 0.003476 | 33 |  |  |  |
| **Actinobacteria** | Treatment | 23.77 | 5 | 4.753 | 1.165 | 0.3507 |
|  | Residual | 114.2 | 28 | 4.079 |  |  |
|  | Total | 138.0 | 33 |  |  |  |
| **Bacteroidetes** | Treatment | 763.3 | 5 | 152.7 | 0.5593 | 0.3507 |
|  | Residual | 3668 | 28 | 131.0 |  |  |
|  | Total | 4431 | 33 |  |  |  |
| **Cyanobacteria** | Treatment | 0.1669 | 5 | 0.03338 | 1.727 | 0.1611 |
|  | Residual | 0.5411 | 28 | 0.01933 |  |  |
|  | Total | 0.7080 | 33 |  |  |  |
| **Deferribacteres** | Treatment | 0.7622 | 5 | 0.1524 | 0.957 | 0.4602 |
|  | Residual | 4.457 | 28 | 0.1592 |  |  |
|  | Total | 5.219 | 33 |  |  |  |
| **Firmicutes** | Treatment | 782.9 | 5 | 156.6 | 1.380 | 0.2617 |
|  | Residual | 3176 | 28 | 113.4 |  |  |
|  | Total | 3959 | 33 |  |  |  |
| **Lentisphaerae** | Treatment | 0.03576 | 4 | 0.007153 | 0.1532 | 0.2119 |
|  | Residual | 0.1307 | 23 | 0.004669 |  |  |
|  | Total | 0.1665 | 27 |  |  |  |
| **Proteobacteria** | Treatment | 6.429 | 5 | 1.286 | 1.832 | 0.1389 |
|  | Residual | 19.65 | 28 | 0.7018 |  |  |
|  | Total | 26.08 | 33 |  |  |  |
| **TM7** | Treatment | 1.746e-005 | 5 | 3.492e-006 | 0.1202 | 0.3336 |
|  | Residual | 8.132e-005 | 28 | 2.904e-006 |  |  |
|  | Total | 9.878e-005 | 33 |  |  |  |
| **Tenericutes** | Treatment | 50.38 | 5 | 10.08 | 0.5833 | 0.7125 |
|  | Residual | 483.7 | 28 | 17.28 |  |  |
|  | Total | 534.1 | 33 |  |  |  |
| **Verrucomicrobia** | Treatment | 6.469 | 5 | 1.294 | 0.8199 | 0.5459 |
|  | Residual | 44.19 | 28 | 1.578 |  |  |
|  | Total | 50.65 | 33 |  |  |  |
| **TM6** | Treatment | 9.994e-005 | 5 | 1.999e-005 | 0.9224 | 0.4812 |
|  | Residual | 0.0006068 | 28 | 2.167e-005 |  |  |
|  | Total | 0.0007067 | 33 |  |  |  |

One-way ANOVA was used to examine week 10 differences between the means of all groups for each specified phylum including sum of squares (SS), degrees of freedom (DF), mean square (MS), “F” values and significant “*p*” values.
